# Repositioning Titanium: An *In Vitro* Evaluation of Laser-Generated Microporous, Microrough Titanium Templates As a Potential Bridging Interface for Enhanced Osseointegration and Durability of Implants

**DOI:** 10.3389/fbioe.2017.00077

**Published:** 2017-12-11

**Authors:** Daniel Tang, Liang-Yo Yang, Keng-Liang Ou, Richard O. C. Oreffo

**Affiliations:** ^1^Centre for Human Development, Stem Cells and Regeneration, Faculty of Medicine, University of Southampton, Southampton, United Kingdom; ^2^Department of Physiology, School of Medicine, College of Medicine, China Medical University, Taichung, Taiwan; ^3^Research Center for Biotechnology, China Medical University Hospital, China Medical University, Taichung, Taiwan; ^4^Department of Biotechnology, College of Medical and Health Science, Asia University, Taichung, Taiwan; ^5^Department of Dentistry, Cathay General Hospital, Taipei, Taiwan; ^6^Department of Dentistry, Taipei Medical University Hospital, Taipei, Taiwan; ^7^Department of Dentistry, Taipei Medical University – Shuang Ho Hospital, New Taipei City, Taiwan; ^8^3D Global Biotech Inc., New Taipei City, Taiwan

**Keywords:** implants, osteoinduction, osseointegration, titanium, skeletal stem cells

## Abstract

Although titanium alloys remain the preferred biomaterials for the manufacture of biomedical implants today, such devices can fail within 15 years of implantation due to inadequate osseointegration. Furthermore, wear debris toxicity due to alloy metal ion release has been found to cause side-effects including neurotoxicity and chronic inflammation. Titanium, with its known biocompatibility, corrosion resistance, and high elastic modulus, could if harnessed in the form of a superficial scaffold or bridging device, resolve such issues. A novel three-dimensional culture approach was used to investigate the potential osteoinductive and osseointegrative capabilities of a laser-generated microporous, microrough medical grade IV titanium template on human skeletal stem cells (SSCs). Human SSCs seeded on a rough 90-µm pore surface of ethylene oxide-sterilized templates were observed to be strongly adherent, and to display early osteogenic differentiation, despite their inverted culture in basal conditions over 21 days. Limited cellular migration across the template surface highlighted the importance of high surface wettability in maximizing cell adhesion, spreading and cell-biomaterial interaction, while restricted cell ingrowth within the conical-shaped pores underlined the crucial role of pore geometry and size in determining the extent of osseointegration of an implant device. The overall findings indicate that titanium only devices, with appropriate optimizations to porosity and surface wettability, could yet play a major role in improving the long-term efficacy, durability, and safety of future implant technology.

## Introduction

Metals, such as stainless steel, cobalt-based alloys, as well as titanium and its alloys, have served as mainstream materials for creating biomedical implants with superior mechanical strength and resilience for use in long-term load-bearing applications, for many years in comparison to biomaterial alternatives such as polymers and bioceramics. To date, titanium and titanium alloys are preferred for biomedical applications, given their high elastic modulus, proven biocompatibility, and established resistance to corrosion and fatigue deformation (Long and Rack, 1998; Xue et al., 2007). The spontaneous formation of a 1.5- to 10-nm-thick native oxide film (TiO_2_), on the surface of titanium (and titanium alloys) upon exposure to air at room temperature has been identified as primarily responsible for the physical and biological properties of titanium (Kirmanidou et al., 2016). However, significant numbers of titanium-based load-bearing implants still fail within 15 years of implantation as a consequence of infection, aseptic loosening, and interfacial instability due to poor bonding between the bone and the implant surface (Goriainov et al., 2014). Stress-shielding caused by a mismatch in modulus between bone and implant (10–30 GPa for cortical bone compared to 120 GPa for titanium alloys) and wear-induced osteolysis due to excessive metal ion release in the implant area (particularly with alloy-based implants) are additional factors that can lead to implant failure in patients (Xue et al., 2007; Kirmanidou et al., 2016).

Given an increasingly aging population worldwide, there has been a rapid expansion of research in the field of biomaterials and scaffold fabrication methods for tissue engineering and regenerative medicine purposes (Hutmacher, 2001), with an increasing trend toward utilizing polymeric, ceramic, and/or organic materials (or a combination thereof) to generate biocompatible scaffolds with varying geometry, porosity, and topography (Ducheyne et al., 2017). In the past decade, there has been growing interest in the use of various additive manufacturing (AM) techniques for the generation of these scaffolds, with tissue biofabrication becoming a major focus of tissue engineering research (Mota et al., 2015; Pedde et al., 2017). Bone tissue engineering is no exception, having adopted biofabrication approaches in a bid to overcome issues with metal implants, and in lieu of the rising need for hip and knee replacements, and total arthroplasty revisions. Current bone tissue engineering approaches have been reviewed in-depth in various publications and will not be elaborated upon in the context of this study (Thibault et al., 2013; Oryan et al., 2014; Tang et al., 2016). A concurrent development has seen an increased use of porous-surfaced and highly porous metallic implants. Their popularity is due to increased clinical success in their use in hip and knee arthroplasties. Ongoing optimizations to fabrication methods to further improve the success of these implants are currently being investigated (Lewallen et al., 2015). One approach has been to utilize surface modification techniques to generate topographical adjustments at a micro- and nano-structural level in the creation of new orthopedic and dental implants. Surface characteristics of biomaterials have previously been shown to exert a critical influence over the speed of osseointegration (Feller et al., 2015; Hotchkiss et al., 2016). Implants that can rapidly osseointegrate enhance implant stability and allow for earlier implant loading and patient mobility (Bosshardt et al., 2017). Such surface modification approaches have been demonstrated in *in vitro* and *in vivo* to facilitate earlier osseointegration between the implant surface and native bone (Lincks et al., 1998; Zinger et al., 2005; Zhao et al., 2007; Gittens et al., 2011; Banik et al., 2016), crucial for healing and successful bone regeneration *in situ*. Approaches to enhance osseointegration include (i) anodic oxidation to enhance the thickness of the naturally occurring oxide layer, (ii) sandblasting, or acid etching to create surface roughness for enhanced cellular adhesion and survival, and wear resistance of titanium implants (Gittens et al., 2014). However, elements within titanium alloys can form other oxides on the alloy surface, reducing the efficacy of treatments (Kirmanidou et al., 2016). A simple alternative approach is scaffold surface coating. Studies utilizing biochemical or biomimetic modification techniques to add collagen type 1 (Morra et al., 2011), alginate hydrogels containing simvastatin (Pullisaar et al., 2013), polytetrafluoroethylene (Fleischmann et al., 2015), and amorphous calcium and phosphorus (Moura et al., 2013) to titanium implants have shown increased osteogenesis of seeded human mesenchymal stem cells (MSCs), human osteoblasts, MG63 cells, and osteoprogenitor cells, respectively.

Increasing surface micro-scale roughness, with feature sizes similar to osteoclast resorption pits (up to 100 µm in diameter) and cell dimensions, can enhance osteoblast differentiation and local factor production *in vitro*, increase bone-to-implant contact *in vivo*, and improve wound healing (Gittens et al., 2011). There remains, however, conflicting evidence regarding the effect of nano-scale surface roughness on osteoblast differentiation, particularly in the concomitant absence of micro-scale surface roughness. Indeed, some studies have attempted to combine both micro- and nano-scale roughness to promote osseointegration (Zhao et al., 2006, 2007). Although initial results appeared to show increased osteoblast proliferation and differentiation, the creation of such tailored surfaces without alteration or modification of other surface characteristics has proved challenging, and separation of nano-scale effects from other surface features, for example, surface chemistry, surface energy, and micro-scale roughness (Gittens et al., 2011) has proved difficult.

A different approach has been the development of porous materials for coating load-bearing implants to enhance bone ingrowth and thus improve implant fixation (Otsuki et al., 2006; Li et al., 2007; Xue et al., 2007; Bandyopadhyay et al., 2017). However, the optimal pore size in facilitating cell infiltration and colonization remains debatable (Itala et al., 2001; Hollander et al., 2006; Otsuki et al., 2006; Xue et al., 2007). Nevertheless, there is a general consensus that increasing the porosity of any device results in decreased mechanical integrity and modulus (Shen et al., 2008). Traditional methods for fabricating porous titanium alloy implants include freeze casting, solid state processing (powder or fiber sintering, metallurgy), electro-deposition, space holder method, and liquid state processing (direct or spray foaming, metal injection molding) (Kirmanidou et al., 2016). Typically, the final product is brittle due to localized stress concentrations at pore walls, and the shape and size of porosity achieved with such techniques random. These limitations can be partially overcome with the use of AM technologies such as selective laser melting (Warnke et al., 2009; Van der Stok et al., 2013), selective electron beam melting, and laser engineered net shaping (Bandyopadhyay et al., 2017). It must be noted that the quality of the generated constructs can vary significantly depending on the design and fabrication parameters, which in turn, are closely related to the type of AM process being used. Furthermore, post-processing steps (such as heat treatment or surface modification techniques) are usually required to reduce thermal stresses within the structures generated and to minimize microstructural changes that occur as part of the layer-by-layer building process (Wang et al., 2016).

Titanium has long been investigated for its biocompatibility and its effect on different cell types such as MG63 cells (Zhao et al., 2011), osteoblasts (Lai et al., 2010), and human MSCs (Banik et al., 2016). However, no previous study has investigated the effect of titanium on human skeletal stem cells (SSCs). Stem cells, in general, are a diverse group of cells that have the capacity for unlimited self-renewal under controlled conditions and have the potential to differentiate into a variety of specialized cell types. The term “mesenchymal stem cell” was originally created to refer to a hypothetical common progenitor of a range of non-hematopoietic, non-epithelial, mesodermal tissue. On the basis of *in vitro* assays and surface phenotyping, it has become widely accepted that MSCs exist in a broad range of postnatal tissues, with a broad spectrum of lineage possibilities such as neural tissue, muscle, and adipose tissue. However, the existence of such a ubiquitous MSC has been subject to criticism in the absence of necessary *in vivo* experimental support (Bianco et al., 2013). The term “skeletal stem cell” has instead been postulated to define self-renewing stem cells from bone marrow stroma that are responsible for the regenerative capacity inherent to bone (Bianco et al., 2013; Dawson et al., 2014). A range of surface markers, which include STRO-1, allows for the prospective, selective isolation of these cells (Tare et al., 2012). Given the challenges faced in improving current implant technology for bone replacement therapy, the potential systemic toxicity of alloys in use in current implants, and that no previous studies involving titanium investigated their effect on SSCs, we have utilized laser-modified microporous, microrough medical grade IV titanium templates to determine how: (i) surface topography, (ii) composition, (iii) wettability, and (iv) pore geometry and size, could influence the cellular behavior of SSCs. Furthermore, we have examined whether such properties could induce osteogenic differentiation of SSCs cultured in basal media. We have inverted the seeded surface of these templates and suspended each inverted template within a culture well to better simulate a three-dimensional culture environment, and determine if such conditions affect cellular adhesion and migration (and therefore osseointegration). Finally, as titanium- and alloy-based surfaces are known to react with their microenvironment, thereby potentially reducing the efficacy and osseointegrative capacity of implants, this study investigated whether methods of sterilization and storage could alter the surface properties of the titanium templates.

## Materials and Methods

### Production of Laser Processed Porous Titanium Templates

Titanium templates (10 mm × 10 mm × 0.1 mm) were manufactured under commercial license by Industrial Technology Research Institute, Taiwan, and provided by Taipei Medical University, Taiwan (Figure S1 in Supplementary Material). Each titanium template was machined in air using an 800 nm wavelength regenerative amplified titanium:sapphire laser (SPITFIRE, Spectra-Physics), operated at a repetition rate of 1 kHz, with a pulse duration of 120 fs. Maximal pulse energy was 3.5 mJ. The laser power was monitored by a detector and adjusted using a half-wave plate and a polarization beam splitter. Irradiation timing was controlled by a mechanical shutter. The machining lens comprised a long working distance 10× objective lens, with 0.26 numerical aperture (M Plan Apo NIR, Mitutoyo). The position of the objective lens could be adjusted in the *z*-axis, and the focused spot diameter used was approximately 5 µm. Micropores were produced by moving the sample on an *x*–*y* mobile stage. The fabrication process was monitored continuously *via* a coaxial machine vision system. 90-µm pores were created on one surface of a medical grade IV titanium sheet using the focused laser beam which bored through the thickness of the material (in a conical fashion), generating 9-µm pores on the under-surface of the 0.1 mm-thick titanium sheet. The pore sizes were chosen to mimic the size of osteoclast resorption pits, which can measure up to 100 µm in diameter. The edges of each template were generated by laser cutting. Fifty templates underwent post-processing ethylene oxide sterilization (EOS) at Taipei Medical University Hospital, Taiwan. Once sterilized, each template was individually vacuum sealed in sterile packaging. Twenty templates were rinsed in an antibacterial, anti-mycotic solution before being exposed to ultraviolet light (UV) for 2 h and air-dried prior to storage at room temperature in a sealed petri dish. Four non-patterned medical grade IV titanium squares were also provided for surface characterization comparison testing purposes.

### Surface Characterization

#### Surface Appearance and Roughness

Qualitative assessments of the macro- and microstructure of each surface were acquired by scanning electron microscopy (SEM, FEI Quanta 200, Thermo Fisher Scientific, USA). Cell-free templates were analyzed without the addition of a conductive coat. The working distance for visualization was between 9.53 and 9.62 mm, with a spot size of 3 nm, and an accelerating voltage of 10 kV under high vacuum conditions.

The surface roughness of each surface of a cell-free EOS template was measured using a microfigure measuring instrument (ET4000A, Kosaka Laboratory Ltd., Japan). Each sample was fixed onto a sample platform and scanned using a 1-µm needle tip, under a 10 μN force and a speed of 5 mms^−1^, for 1 mm in length. Roughness values (arithmetic average roughness: *R*_a_ and geometric average roughness: *R*_q_) were calculated from measurements performed at three different points per sample. In order to determine whether the manufacturing process resulted in changes to the surface roughness, similar measurements were carried out on non-patterned medical grade IV titanium (cut to the same dimensions).

#### Surface Chemical Composition and Wettability

A JEOL JSM-6500F SEM (JEOL Japan, Inc., Japan) with a Si(Li) detector was used to perform energy-dispersive X-ray spectroscopy (EDS), which determined the surface composition of non-patterned medical grade IV titanium, EOS, and UV titanium templates. As the tested templates contained no biological material, no fixation or drying steps were required. Each template was fixed onto a copper stub and electrically grounded by carbon coating, before each surface was sputter-coated with an ultra-thin platinum film. Imaging was then performed in a high vacuum using a 10 kV electron beam and working distance of 15 mm. Single-point measurements and mapping analyses were performed using INCAEnergy software (Oxford Instruments, UK). Each surface characterization procedure was performed on six regions of two areas per surface type, and the average reading from two templates was calculated.

Surface wettability was measured by performing contact angle testing using a GBX Digidrop-DI goniometer and its accompanying Visiodrop software (GBX, Ireland). A deionized water droplet volume of 1.5 µl was used for every measurement. Point selection was repeated five times per position, of which three were selected for each template surface.

### Isolation, Culture, and Seeding of Human SSCs

As previously detailed in the protocol by Tare et al. (2012), SSCs were isolated from human bone marrow samples from two male patients (aged 66 and 67) and a female patient (aged 70) following hip arthroplasty, with full written, informed patient consent, and ethical approval (NHS England Local Research Ethics Committee, number 194/99). Briefly, isolated SSCs were cultured in 25 ml of basal media comprising of αMEM (Lonza, Switzerland), 10% v/v fetal calf serum (Thermo Fisher Scientific, USA) and 1% v/v penicillin (10,000 units), streptomycin (10 mg/ml) (Lonza, Switzerland), per 175 cm^2^ Corning^®^ cell culture flask, for 10 days post-isolation in a humidified, 37°C, 5% CO_2_ incubator (NuAire, UK). The culture media was changed every three to four days. Cells were passaged at 70–80% confluency and all experiments undertaken with cells at passage 1 or 2. Cell counts were performed using a Fast-Read 102^®^ disposable counting slide (Dutscher Scientific, UK).

Titanium templates were seeded by manually pipetting 100 µl of basal media containing 1.5 × 10^4^ SSCs directly onto the center of each template surface. Placing the cell droplet centrally allowed for the observation of cell migration across the template surface during the culture period. This was followed by a 30 min incubation of the seeded templates in a petri dish placed in a humidified, 37°C, 5% CO_2_ incubator. The seeded template surface was inverted prior to being suspended within the well of a Corning^®^ 12-well plate by a custom-made well insert device. EOS templates were cultured for 1, 3, 7, 14, and 21 days in basal media (*n* = 3 per time-point). A similar number of SSCs per well (*n* = 3 per time-point) were cultured in basal and osteogenic media [basal media supplemented with 25 nM 1,25-dihydroxyVitaminD_3_ and 100 µM ascorbic acid-2-phosphate (both Sigma Aldrich, UK)] on tissue culture polystyrene plastic (TCP) as comparative controls.

### Human SSC Response and Functionality

#### Immunocytochemical Staining

Seeded templates from days 7 and 14 were fixed in 4% paraformaldehyde for 15 min and permeabilized with 0.5% Triton X-100 in PBS for 20 min. The templates were then blocked with 5% goat serum (Sigma Aldrich, UK) and 0.5% Triton X-100 in PBS for 1 h prior to incubation at 4°C overnight with mouse IgG1 anti-alpha tubulin antibody (1:100; Thermo Fisher Scientific, USA) diluted in 1% bovine serum albumin and 0.5% Triton X-100 in PBS. The following day, these templates were incubated with Alexa-Fluor 488-conjugated IgG goat anti-mouse secondary antibody and TRITC-conjugated phalloidin which stains for F-actin (both 1:100; 1 h at room temperature; Merck Millipore, USA). This was followed by nuclear counterstaining with DAPI (1:250 in PBS; 5 min; Sigma Aldrich, UK). The final step involved incubation of the templates with HCS CellMask™ Deep Red stain (2 µl per 10 ml PBS; 30 min; Thermo Fisher Scientific, USA), which stained the cytoplasm of the seeded cells, allowing for the visualization of their morphology. Stained templates were kept in PBS prior to imaging using a Leica SP5 Confocal Microscope. Three-dimensional image reconstructions were done using the Leica Application Suite X software.

#### RNA Isolation and Quantitative Reverse Transcription Polymerase Chain Reaction (RT-qPCR) Analysis

Seeded templates (*n* = 3) were lysed with TRIzol^®^ reagent (Thermo Fisher Scientific, USA) at day 7, 14, and 21 end-points. RNA was extracted from cell lysates by adding 200 µl of chloroform per 1 ml of TRIzol^®^ in a fume hood. The mixtures were vortexed for 30 s to ensure adequate mixing before incubating them for 3 min at room temperature. The mixtures were then centrifuged at 11,000 × *g* for 15 min at 4°C in a Heraeus Biofuge fresco centrifuge (Thermo Fisher Scientific, USA). The clear supernatant was transferred to an ISOLATE II filter column, a part of the ISOLATE II RNA Mini Kit (Bioline, UK). RNA purification was then performed according to the manufacturer’s guidelines. RNA concentrations were measured using a NanoDrop spectrophotometer (Thermo Fisher Scientific, USA). cDNA synthesis was carried out using a SuperScript VILO™ kit (Thermo Fisher Scientific, USA), according to the manufacturer’s recommendations. An Applied Biosystems 7500 Real-Time PCR System, with PowerSYBR^®^ Green PCR Master Mix (both Thermo Fisher Scientific, USA) were used to perform RT-qPCR for Runt-related transcription factor 2 (Runx2), collagen type 1 alpha 1 chain (Col1a1), alkaline phosphatase (ALP), osteopontin (OPN), osteocalcin (OCN), and β-actin, with TATA-box binding protein (TBP) as the housekeeping gene reference. All primers (all Sigma Aldrich, UK) used are listed in Table S1 in Supplementary Material. RT-qPCR analysis was performed in triplicate at days 7, 14, and 21. Relative mRNA levels were calculated using the comparative CT method, and normalized to TBP. The derived relative expression of each gene marker at all time-points was then normalized against the relative expression of the same marker from the basal control day 7 samples.

#### Cell Viability, Proliferation, and Functionality

To determine seeded cell viability, triplicate seeded templates were exposed to 0.4 mM Calcein AM and 2 mM ethidium homodimer-1 (Thermo Fisher Scientific, USA) for 1.5 h in a 37°C incubator at days 1, 3, 7, 14, and 21. A Nikon Eclipse T*i* microscope fitted with a FITC filter was used to capture images of live and/or dead seeded SSCs.

Skeletal stem cell proliferation at days 3, 7, 10, 14, and 21 was determined using a WST-1 cell proliferation assay (Roche, Switzerland), as per the manufacturer’s protocol. A 1:10 dilution of the reagent to media was used for incubating the templates over a 3 h period. 100 µl of the resultant mixture was plated in triplicate for each test sample in addition to a control consisting of basal media and the WST-1 reagent only (which was incubated under the same conditions). A Varioskan^®^ Flash microplate reader (Thermo Fisher Scientific, USA) was used to read the corresponding optical densities at 420 nm, with a reference wavelength reading at 610 nm.

The interaction between the seeded SSCs and the template surface topography was qualitatively examined using a FEI Quanta 200 SEM at days 7, 14, and 21. All samples were fixed in 3% glutaraldehyde/4% formaldehyde in pH 7.2 phosphate buffer solution at 4°C overnight. The fixed samples were dehydrated in an ethanol series (30, 50, 70, 95, and 100%) followed by a critical point drying procedure. The samples were coated with a 7-µm gold-palladium film. The working distance for visualization was between 10.66 and 11.83 mm, with a spot size of 4–4.5 nm, and an accelerating voltage of 10 kV under high vacuum conditions.

Triplicate culture supernatants were collected at days 3, 7, 10, 14, and 21 for ALP activity analysis using a colorimetric assay (Abcam, UK), according to the manufacturer’s instructions. Culture media was changed 24 h prior to each assay to ensure that the ALP activity measured was over a 24-hour period. This assay kit utilizes p-nitrophenyl phosphate as a phosphatase substrate, which turns yellow when dephosphorylated by ALP. Following a 1-h incubation in the dark in a 25°C incubator, the optical density of the samples and standards was read using a Varioskan^®^ Flash microplate reader (Thermo Fisher Scientific, USA) at an absorbance wavelength of 405 nm. The results are presented as calculated activity values based on the absolute absorbance readings obtained, with no normalizations performed.

### Statistical Analysis

Values are presented as means ± SD. Data shown in figures is representative of one of the triplicate experiments performed. Statistical analysis was performed using GraphPad Prism version 7.00 for Windows (GraphPad software, La Jolla, CA, USA, www.graphpad.com). Two-way analysis of variance (ANOVA), followed by *post hoc* testing, was conducted on the influence of culture time and culture approach on cellular proliferation, ALP activity and gene expression. Culture time groups consisted of day 7, 14, and 21 end-points. Culture approach comprised of basal media, osteogenic media, and EOS titanium groups. *Post hoc* analysis provided information about which levels within each variable were significant, with a *p* value less than 0.05 taken to indicate a statistically significant difference.

## Results

### Surface Appearance and Roughness

**Figure 1:**
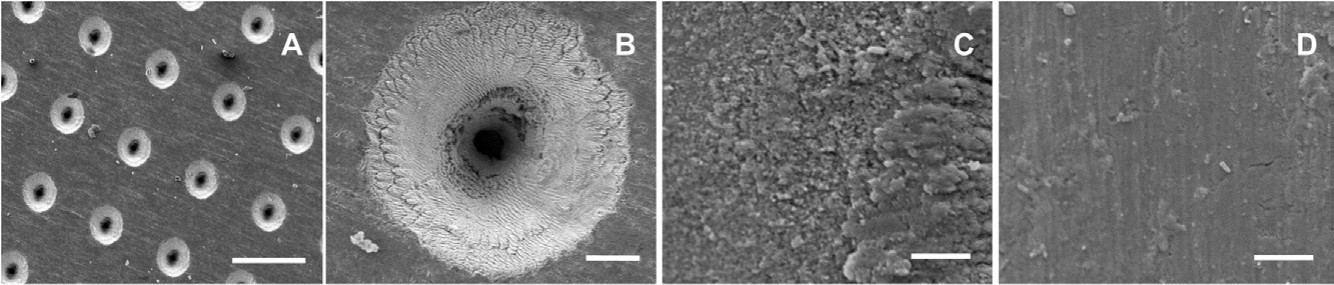
Surface appearance of cell-free titanium templates using scanning electron microscopy. The images demonstrate the different surface topographies across the template surface. **(A)** An overview of the 90-µm pore surface (scale bar represents 200 µm). **(B)** A higher magnification view of a 90-µm pore (scale bar represents 20 µm). **(C)** Surface roughness is evident at a microscale level within the vicinity of the 90-µm pore (scale bar represents 5 µm). **(D)** The interpore area demonstrates a much smoother appearance (scale bar represents 5 µm).

Scanning electron microscopy image analysis revealed that the laser process created templates with dual surfaces consisting of a uniformly porous pattern (Figure 1A; Figure S2A in Supplementary Material). These regularly aligned pores were conical in shape (Figure 1B). On the upper surface of a template, the pore diameter measured 90.9 ± 2 µm, with an interpore distance of 203.3 ± 1.9 µm, while pore diameter on the under-surface measured 9.4 ± 1.1 µm. High magnification SEM images showed that the 90-µm pores had a coarse surface appearance when compared to those of the 9-µm pores or the interpore areas of both template surfaces (Figures 1C,D; Figures S2C,D in Supplementary Material). This difference in surface roughness was confirmed by surface roughness measurements, which demonstrated the 90-µm pore surface had a *R*_a_ of 2.21 ± 0.27 µm, and a *R*_q_ of 3.21 ± 0.34 µm, with the interpore areas (indicated by black arrows) demonstrating *R*_q_ values within a 1 µm range (Figure S3C in Supplementary Material). The 9-µm pore surface displayed a *R*_a_ of 0.26 ± 0.04 µm and a *R*_q_ of 0.4 ± 0.03 µm (Figure S3D in Supplementary Material). The laser process increased the surface roughness of non-patterned medical grade IV titanium, as evidenced by surface roughness measurements of the upper-surface that revealed a *R*_a_ of 0.16 ± 0.02 µm and a *R*_q_ of 0.21 ± 0.02 µm (Figure S3A in Supplementary Material) while the under-surface displayed a *R*_a_ of 0.09 ± 0.01 µm and a *R*_q_ of 0.12 ± 0.01 µm (Figure S3B in Supplementary Material).

### Surface Chemical Composition and Wettability

EDS surface mapping revealed that non-patterned medical grade IV titanium controls comprised of 90.2 wt% titanium, 2.7 wt% carbon, and 7.1 wt% platinum. No measurable level of oxygen was detected. EDS surface mapping revealed that EOS titanium templates comprised of 65.8 wt% titanium, 21.6 wt% oxygen, 5.7 wt% carbon, and 6.9 wt% platinum, with the highest concentration of oxygen localized to the immediate vicinity within the 90-µm pores (Figure S4 in Supplementary Material). Focal chemical composition measurements of the 90-µm pore area indicated an average oxygen content of 44.2 ± 3.6 wt% and an average titanium content of 54.1 ± 5.8 wt%. The interpore areas of both surfaces demonstrated no measureable levels of oxygen while the 9-µm pore areas consisted of 33.4 ± 10.9 wt% oxygen and 65.3 ± 9.8 wt% titanium (data not shown). The data indicate the presence of a greater amount of titanium dioxide on the 90-µm pore surface. EDS surface mapping of the 90-µm pore surface of UV titanium templates revealed the surface was composed of 70 wt% titanium, 18.1 wt% oxygen, 6.4 wt% carbon, and 5.5 wt% platinum, demonstrating 3.5 wt% reduction in oxygen content in comparison to the 90-µm pore surface of EOS templates (Figure S4 in Supplementary Material).

The 90-µm pore surface of EOS titanium templates displayed a contact angle of 87.6 ± 8°, while the 9-µm pore surface had a contact angle of 83.3 ± 5.3°, indicating that both surfaces were poorly hydrophilic. Remarkably, the 90-µm pore surface of UV titanium templates had a statistically significant larger contact angle of 112.6 ± 0.9°, which worsened to 118.2 ± 1.5° after 2 months of storage in air at 25°C, indicating the hydrophobicity of the UV titanium surface increased as the exposed titanium surface interacted with atmospheric particles during storage.

### Cell Adhesion and Immunocytochemical Staining

**Figure 2:**
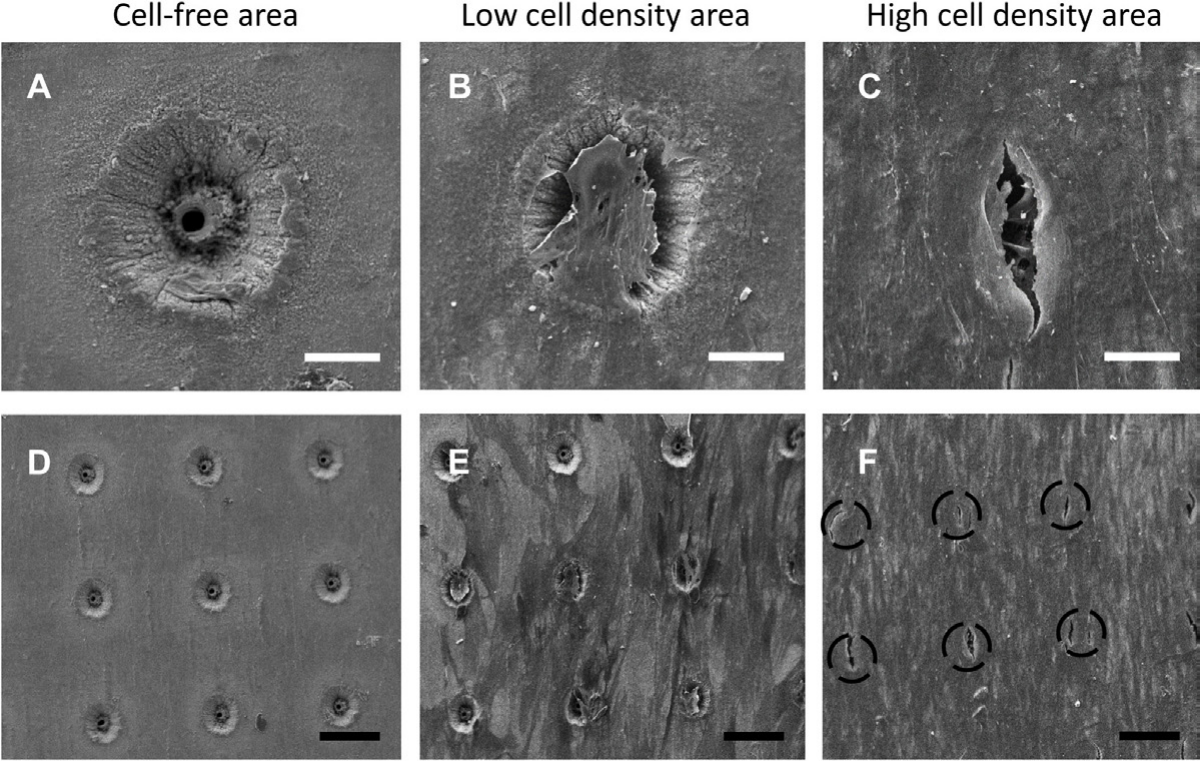
Scanning electron microscopy (SEM) images of skeletal stem cell (SSC)-seeded ethylene oxide sterilization (EOS) titanium scaffolds at day 14 demonstrating the variation in cell density in different areas of a template, in addition to the effect of cell density on cell behavior on the template surface and pore areas: **(A)** 90-µm pore, no cells (scale bar represents 35 µm). **(B,C)** 90-µm pore appearance as cell density increases (scale bar represents 40 µm), demonstrating cells bridging the pores. **(D)** 90-µm pore surface without cells (scale bar represents 130 µm). **(E,F)** 90-µm pore surface appearance as cell density increases (scale bar represents 150 µm). Black dashed circles mark the position of 90-µm pores.

**Figure 3:**
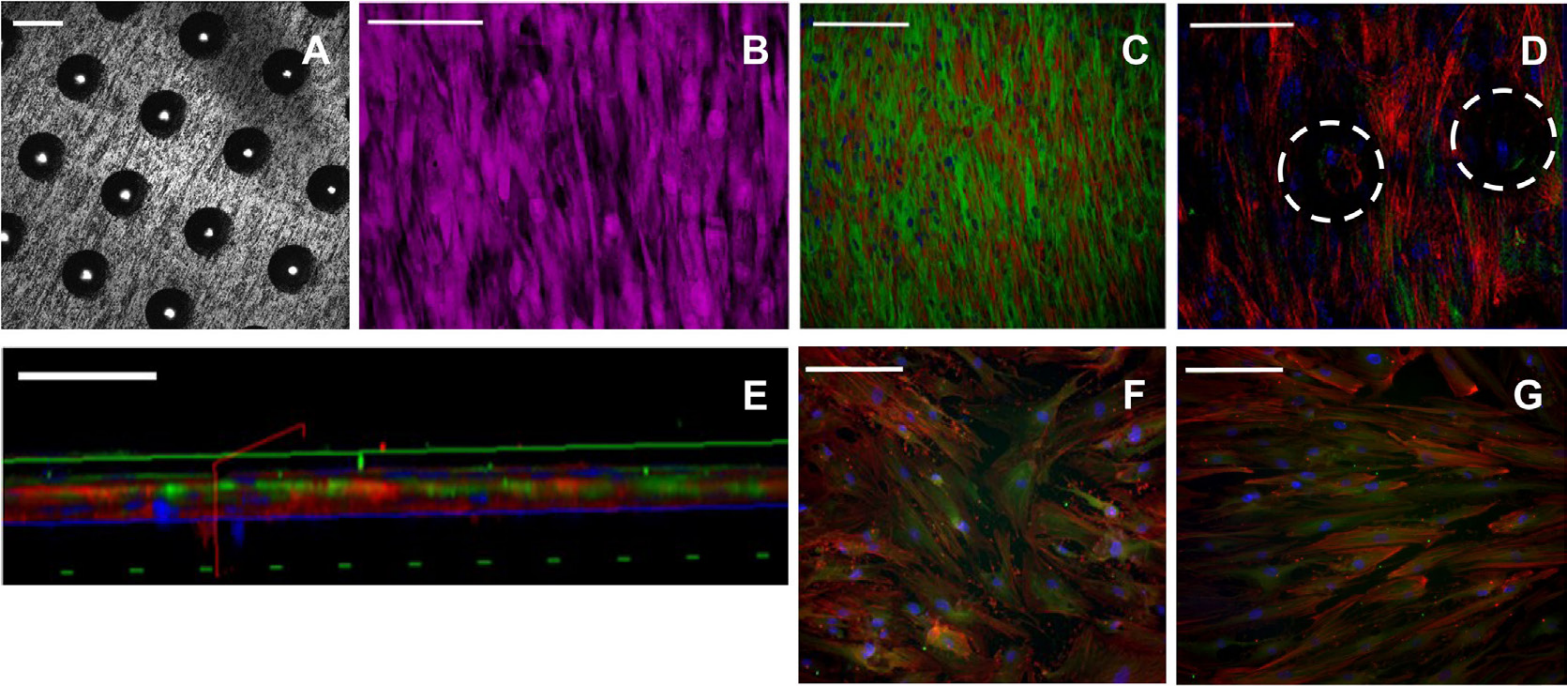
Immunocytochemical staining of skeletal stem cells (SSCs) seeded on ethylene oxide sterilization (EOS) titanium templates and tissue culture plastic (TCP) at day 14. Confocal microscopy images of the 90-µm pore surface of the titanium template using **(A)** reflectance mode (scale bar represents 100 µm), **(B)** normal mode, showing HCS CellMask™ Deep Red stained SSCs (magenta) seeded on the 90-µm pore surface of the titanium template (scale bar represents 100 µm). **(C)** Cytoskeletal filament orientation and cell distribution as demonstrated by F-actin (TRITC—red), α-tubulin (AlexaFluor-488—green), and nuclei (DAPI—blue) stains, scale bar represents 200 µm. **(D)** F-actin, α-tubulin, and nuclei appearance of SSCs directly in contact with the titanium surface, and SSCs growing within the 90-µm pores (white dotted circles). Scale bar represents 100 µm. **(E)** Virtual cross-sectional view of **(D)**. The solid blue axis delineates the template surface. Scale bar represents 50 µm. **(F,G)** Cytoskeletal filament orientation of SSCs cultured on TCP in basal media and osteogenic media, respectively. Scale bar represents 200 µm.

Scanning electron microscopy image analysis demonstrated SSCs adherent on the seeded 90-µm pore surface (Figure 2; Figure S5 in Supplementary Material), despite the short incubation time for cell attachment, and the seeded surface being inverted in each well. Despite the similarity in the surface wettability of the 9 µm and 90 µm pore surfaces, the 9 µm pore surface demonstrated poor cell adhesion and minimal cell proliferation (data not shown). HCS CellMask™ Deep Red staining (shown in magenta) confirmed that seeded SSCs developed an increasingly stellate-shaped morphology over time, as well as a compact and organized aligned orientation of SSCs within high cell density areas on the template surface (Figure 3B). Phalloidin staining demonstrated well-defined localization of F-actin (shown in red, Figures 3C–G) to the apical sides of the cytoplasm in SSCs, while α-tubulin (shown in green, Figures 3C–G) extended uniformly from the nucleus throughout the cytoplasm of SSCs. F-actin and α-tubulin fluorescence signal intensity were noted to increase with culture time and were highest in SSCs seeded on EOS titanium templates (irrespective of cell density). Similarly organized F-actin and α-tubulin filament orientation was observed within cells within the topmost layer (Figure 3D; Video S1 in Supplementary Material). However, confocal microscopy revealed that F-actin filament orientation of SSCs in direct contact with the template surface was not as organized or uniform. Furthermore, F-actin filaments of SSCs growing within the 90-µm pores were noted to be contracted and condensed (Figure 3; Video S1 in Supplementary Material). α-tubulin fluorescence signal intensity of these cells increased over time in culture (Figure S6 and Video S1 in Supplementary Material). SSCs cultured on TCP showed fewer F-actin and α-tubulin filament aggregates, while filament orientation was also markedly less uniform (Figures 3F,G). The increased F-actin and α-tubulin fluorescence signal intensities in SSCs seeded on EOS titanium templates indicated greater cytoskeletal activity in response to stimulation by the EOS titanium template surface topography and surface chemistry, resulting in maintained cellular adhesion despite the culture conditions. Cell confluency severely impeded nuclear and cytoskeletal shape analysis quantification as a function of days in culture at the time points performed, and as such, the data have not been included in this study.

**Figure 4:**
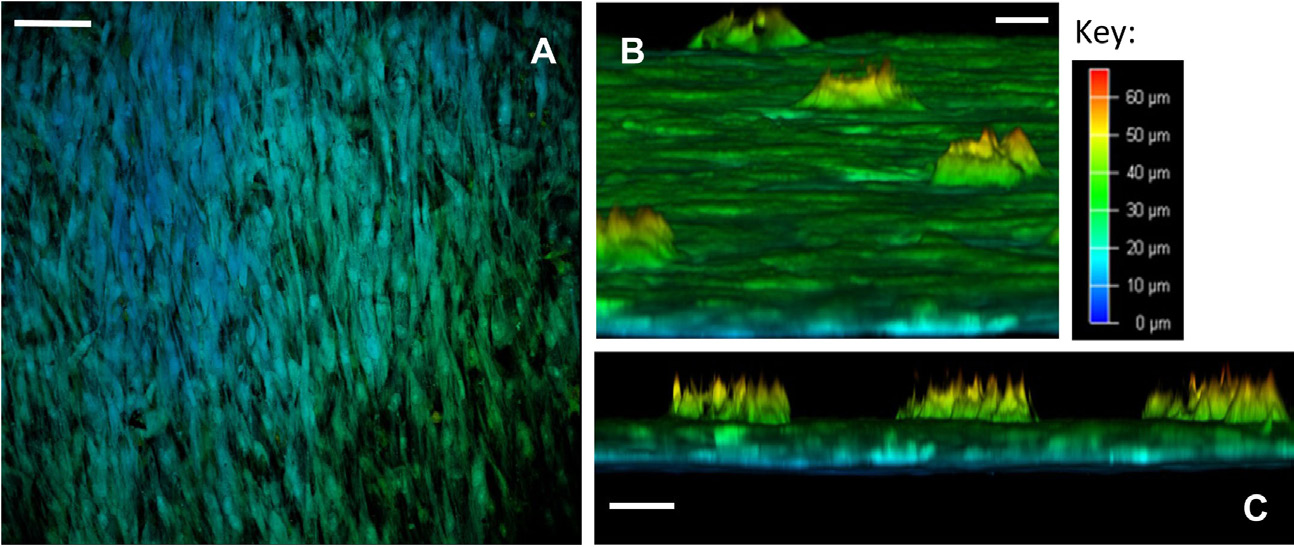
Confocal microscopy depth imaging of skeletal stem cells (SSCs) seeded on ethylene oxide sterilization (EOS) titanium templates at day 14. **(A)** Depth imaging revealed the presence of different cell layers covering the template surface. The presence of blue-labeled cells in areas of higher cell density and green-labeled cells in areas of lower cell density indicates cell layer thickness varied by 35 µm on the template surface, depending on cell density (scale bar represents 100 µm). **(B)** Virtual cross-sectional view of the 90-µm pore surface of the template as cultured: SSCs can be seen growing within the pores up to a depth of 25 µm (scale bar represents 20 µm). **(C)** Virtual cross-sectional view of the titanium template demonstrating the thickness of the cell layer on the template surface, in addition to the uniformity of SSC growth within the 90-µm pores. Note that SSCs growing within the pores fail to completely fill the pore space (scale bar represents 50 µm).

Confocal microscopy was undertaken to perform depth image analysis of the cell layers. Leica Application Suite X software was utilized to build a three-dimensional reconstruction of the template, enabling a virtual cross-section analysis of the template (up to a 70-µm depth). Depth image analysis of high cell density areas revealed a two to three cell thick layer on the seeded surface of the template, measuring up to 35 µm (Figure 4). Less confluent cell areas showed only an adherent monolayer of cells, up to 10-µm thick. Virtual cross-section reconstruction revealed pore bridging in areas of high cell density. However, SSCs were only able to grow 25 µm into the 100 µm deep conical pores, and these cells failed to completely fill the pore space (Figures 4B,C, and supporting video S1).

### RT-qPCR Analysis

**Figure 5:**
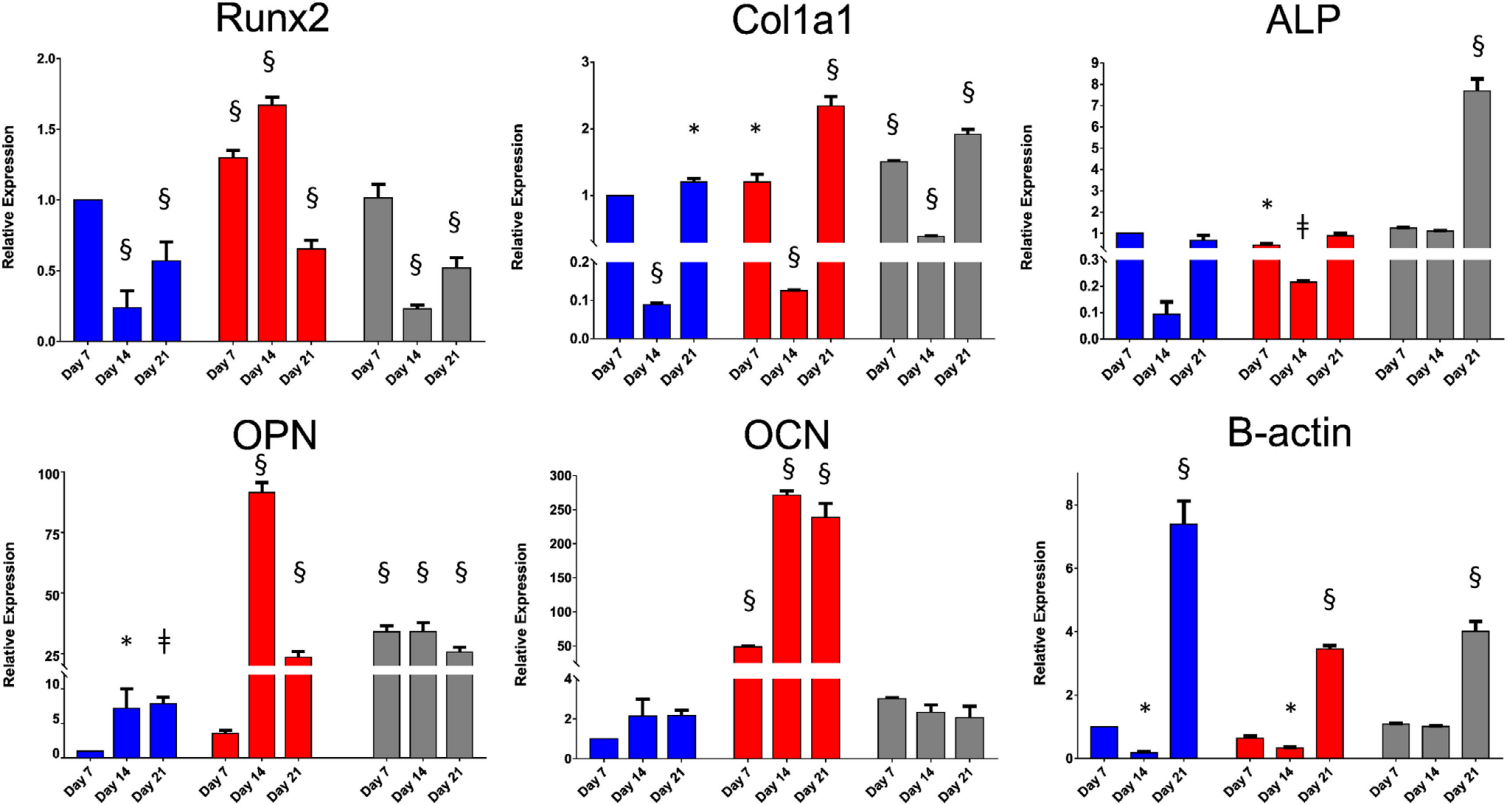
Relative expressions of Runx2, Col1a1, alkaline phosphatase (ALP), osteopontin (OPN), osteocalcin (OCN), and β-actin at days 7, 14, and 21. Skeletal stem cells (SSCs) on TCP were cultured in basal (in blue) and osteogenic (in red) media. SSCs on ethylene oxide sterilization (EOS) templates were cultured in basal media (in gray). Relative expression of each gene marker at all time-points was normalized against the relative expression of the same marker from basal day 7 samples (*p < 0.05, ^ǂ^*p* < 0.01, ^§^*p* < 0.001).

The derived relative expression of each tested gene marker at all time-points was normalized against the relative expression of the same marker from basal control day 7 samples. TBP was chosen as the housekeeping reference gene as previous studies have shown that β-actin expression changes significantly in three-dimensional culture (Rauh et al., 2015). Two-way ANOVA, followed by Dunnett’s multiple comparison test, was performed to determine the effect of culture time and culture approach on gene expression of Runx2, Col1a1, ALP, OPN, OCN, and β-actin. The results are summarized in Table S2 in Supplementary Material. The relative gene expression data are summarized in Figure 5. Although culture time and culture approach were shown to have a significant effect on gene expression in general, the extent of interaction between these two variables was extremely significant for all gene markers tested. Runx2, the earliest marker of osteogenic differentiation, was significantly downregulated by day 14 in SSCs seeded on EOS titanium templates. Col1a1 expression was significantly upregulated at day 7, while ALP expression was very significantly elevated in the titanium group at day 21. This elevated ALP expression indicated that seeded SSCs on EOS titanium were depositing extracellular matrix and initiating matrix mineralization. OPN gene upregulation in the titanium group was initially enhanced at day 7 and remained significantly elevated at days 14 and 21. The degree of increased expression was comparable to SSCs on TCP maintained in osteogenic media. OCN expression was increased in the titanium group, but to a lesser, non-significant extent. Increased OPN (and OCN expression) indicated that SSCs seeded on EOS titanium were induced by the template to undergo osteogenic differentiation, albeit to a lesser extent than SSCs cultured in osteogenic media. A highly significant increase in β-actin expression by day 21 in all three groups was observed, indicating that β-actin expression increased as culture time progressed. Although similar levels of upregulation of Runx2 and Col1a1 expression in SSCs seeded on UV titanium templates were observed, there was reduced (and less persistent) upregulation of OPN and OCN expression, indicating SSCs were less induced to differentiate by UV-irradiated titanium (Figure S7 in Supplementary Material).

### Cell Viability, Proliferation, and Functionality

**Figure 6:**
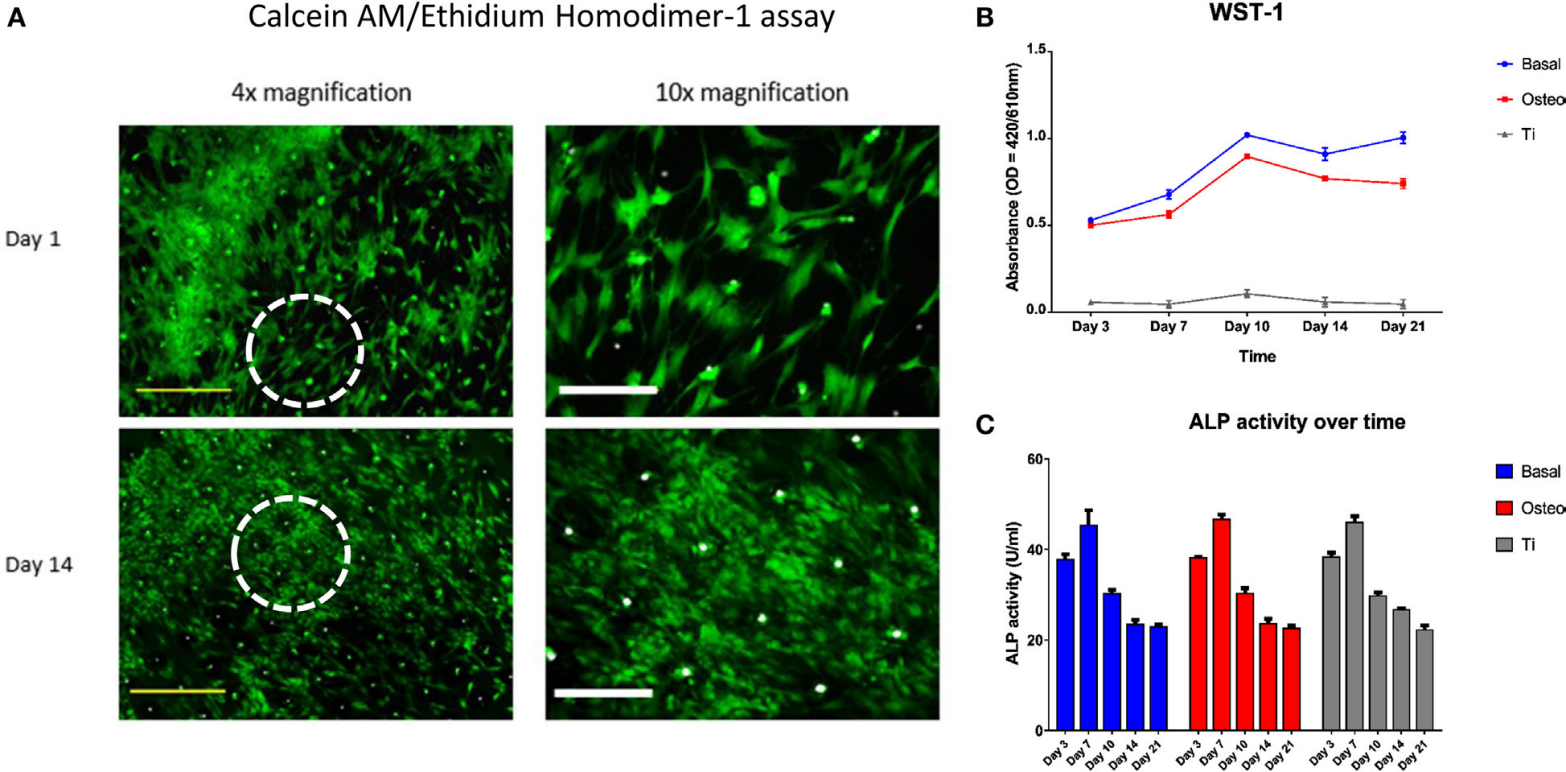
Skeletal stem cell (SSC) viability, proliferation, and functionality over time. **(A)** Merged Calcein AM, Ethidium homodimer-1, and Brightfield images of SSCs on ethylene oxide sterilization (EOS) templates at days 1 and 14 demonstrated good cell viability over time, with a concomitant increase in cell density and stellate morphology. Dotted white circles indicate the area visualized under 10× magnification. Yellow scale bar represents 500 µm, and white scale bar represents 200 µm. **(B)** WST-1 proliferation assay demonstrated a higher proliferative rate of SSCs on TCP when compared to EOS templates over the 21 day culture period (*p* < 0.001). SSCs cultured in osteogenic media had a lower proliferative rate compared to SSCs cultured in basal media between days 7 and 21 (*p* < 0.001), reflecting osteogenic differentiation under 1,25-VitD_3_ induction. **(C)** Alkaline phosphatase (ALP) activity peaked at day 7 in all three groups, with an expected stepwise reduction over the following 2 weeks of culture.

Excellent cell viability and negligible cell death of seeded SSCs over the 21-day culture period on the templates was evidenced by strong Calcein AM and a negative Ethidium homodimer-1 staining (Figure 6A). However, the WST-1 assay revealed a markedly reduced cellular proliferation of seeded SSCs on the EOS titanium templates at all time-points over the 21 day period in comparison to SSCs cultured on TCP in both basal and osteogenic media. Maximal cellular proliferation occurred at day 10 in all groups (Figure 6B). The observations above carried across repeat experiments. SSCs cultured in osteogenic media demonstrated a reduction in cellular proliferation after seven days of culture when compared to SSCs cultured in basal media. Two-way ANOVA followed by Tukey *post hoc* test demonstrated an extremely significant interaction [*F*(8, 24) = 120, *h*^2^ = 0.048] between culture time [*F*(4, 24) = 504.6, *h*^2^ = 0.01] and culture approach [*F*(2, 6) = 1886, *h*^2^ = 0.849] in affecting cellular proliferation (*p* < 0.001), making the interpretation of the significance of the effect of each variable difficult.

Seeded SSCs demonstrated a spindle-like morphology within 24 h of culture, and most progressed to a stellate morphology by day 14 (Figure 6A), irrespective of cell density. SEM image analysis and Calcein AM staining highlighted the variation in cell density on the template surface, whereby the highest density of cells was localized to the immediate vicinity of the droplet area. In general, cell density decreased from the center to the edge of the templates (Figure 2; Figure S5 in Supplementary Material). This pattern emerged by day 3 of culture, and remained unchanged at day 21, suggesting limited cellular migration across the template over the culture period. SSCs were observed to commence formation of colony clusters by day 3, and collectively organized in a similar direction in highly confluent areas by day 14, forming sheet-like cell layers containing minimal intercellular gaps across the template surface, with most 90-µm pores in these areas appearing to be bridged by cells (Figure 2). In low confluency areas, cellular orientation appeared disorganized and random, with areas of the template remaining visible. However, SSCs were still noted to grow into the 90-µm pores, with modest bridging of the 90-µm wide gap of each pore by day 7 (Figure S5 in Supplementary Material).

Figure 6C illustrates the ALP activity of the three groups over the 21-day period. ALP activity reached peak values at day 7 in all three groups, before falling to similar levels over the next 14 days. Two-way ANOVA, followed by Tukey multiple comparison test, revealed that the main effect for time groups yielded an F ratio of *F*(4, 24) = 524.99, *p* < 0.001, *h*^2^ = 0.979, indicating that culture time had a significant effect on ALP activity. However, the main effect for culture approach yielded an *F* ratio of *F*(2, 6)= 1.17, *p* > 0.05, *h*^2^ = 0.001, indicating that culture approach was not significant. The interaction effect was not significant, *F*(8, 24)= 1.61, *p* > 0.05, *h*^2^ = 0.006. Tukey *post hoc* analysis revealed no significant differences in ALP activity between the culture groups at each time point except at day 14, where ALP activity remained significantly higher in the EOS titanium group in comparison to the other two groups (basal-osteogenic = non-significant, basal-titanium = *p* < 0.01, osteogenic-titanium = *p* < 0.05) (Figure S8 in Supplementary Material). Coupled with WST-1 results showing a markedly lower cell proliferation rate in the EOS titanium group and higher ALP gene expression over time in SSCs seeded on EOS titanium, the results indicate enhanced ALP production by SSCs cultured on EOS titanium.

## Discussion

Given the established biocompatibility of titanium, and the associated long-term health issues of utilizing alloys in implants, this study examined whether commercially available medical grade IV titanium could be re-purposed as a bridging interface. We demonstrate that laser-generated microporous, microrough titanium templates facilitate rapid SSC adhesion, and induce early osteogenic differentiation of SSCs throughout a 21-day culture period. Cellular interaction with biomaterials is crucial for the successful, long-term implantation of any medical device. Altering surface roughness can increase surface hydrophobicity, but overall, modulation of surface roughness improves cell adhesion through its effects on adhesion proteins (Olivares-Navarrete et al., 2008; Gittens et al., 2011). Studies by Zinger et al., Zhao et al., Olivares-Navarrette et al., and Banik et al. indicate that microporosity (Zinger et al., 2005), micro-scale roughness (Zhao et al., 2006; Banik et al., 2016), low skewness and low kurtosis topography (Olivares-Navarrete et al., 2014), and high surface energy (Zhao et al., 2005, 2007; Lai et al., 2010) can synergistically enhance osteogenic differentiation of seeded human osteoblast-like or MSCs. High surface energy has also been shown to improve angiogenesis (Raines et al., 2010).

Surface composition is a key factor in determining the characteristic of any scaffold and has been postulated to play a role in the cytocompatibility and osteoinductive effects of a biomaterial on seeded cells. Fabrication methods, as well as surface modification techniques (including biochemical modification), can alter the surface composition, which in turn, can positively, or adversely, affect the performance of an implant *in vivo* (Kirmanidou et al., 2016). It is therefore crucial to elucidate a scaffold composition beforehand, in addition to understanding the effects that modification and manufacturing techniques have on surface properties. Lincks et al. (1998) had demonstrated that MG63 cells underwent greater differentiation when cultured on pure titanium, rather than titanium alloy surfaces. One of the most commonly used titanium alloys in implants, Ti6Al4V, on degradation through wear and corrosion, produces wear debris *in vivo* consisting of aluminum and vanadium particles that have been linked to neurotoxicity, impaired bone remodeling, and osteoblast toxicity. With other metals such as chromium and cobalt also demonstrating toxic effects *in vivo* (Sansone et al., 2013), efforts have been made to identify new alloy combinations for use in medical implants. Ikarashi et al. (2005) utilized a titanium alloy containing 50% zirconium, which showed better cytocompatibility than pure titanium and chromium implants. Additionally, alloy elements form different oxides apart from titanium oxide on the surface of implants, which could limit the formation of apatite, reducing the osseointegrative capability of the implant (Kirmanidou et al., 2016).

In theory, pure titanium surfaces exhibit high surface energy due to their native oxide layer. However, such surfaces adsorb inorganic anions or organic hydrocarbons within seconds of exposure to the atmosphere, altering their surface chemical composition and reducing hydrophilicity (Fernández-Rodríguez et al., 2014). This has even been reported to cause reversion to the original hydrophilicity of the material, irrespective of surface treatment (Fernández-Rodríguez et al., 2014). Sterilization methods using ethanol or autoclaves are known to further increase the hydrophobicity of a material (Zhao et al., 2005). In our study, the different modes of sterilization used, EOS and UV, resulted in differing oxygen content, with the EOS process generating an increased oxide presence, as demonstrated by EDS mapping (Figure S4 in Supplementary Material). The oxidizing effect of EO gas could result in the increased oxygen content of EOS templates. The increased presence of titanium dioxide could have improved the wettability (and indirectly, the surface energy) of EOS templates, which in turn would enable better cell adhesion. These findings indicate the importance of understanding methods of sterilization and storage as interaction with the atmosphere (once removed from their protective packaging), can potentially, at least in titanium implants, reduce their efficacy.

In general, cells prefer hydrophilic surfaces for adhesion and proliferation (Gittens et al., 2014). However, the contact angle method used to measure the wettability of a material surface becomes less reliable when performed on porous substrates. Potential confounding factors such as air trapping within the pores, high scaffold porosity, and surface concavity or convexity can affect liquid dispersion across the surface, consequently producing inaccurate results. Although the values obtained predicted poor cell adhesion and despite the inverted culture method over the 21-day period, good SSC adhesion on 90-µm pore surfaces was observed (Figures 2 and 3; Figures S5 and S6 in Supplementary Material). Given the 90-µm pore surface consisted mainly of pores, rather than a solid, flat titanium surface, air trapping within the pores could possibly have increased the contact angle readings obtained for both EOS and UV templates. Smaller pores on the 9-µm pore surface would have had less impact on contact angle measurements. The poor hydrophilicity, taken together with lower *R*_a_ and *R*_q_ values may account for the poor cell adhesion observed for the 9-µm pore surface. The strength of cell adhesion was confirmed by increased cytoskeletal activity in SSCs seeded on EOS templates over time (Figure 3; Figure S6 in Supplementary Material) and early and persistent upregulation of β-actin (Figure 5), indicating that 90-µm pore surfaces of EOS templates had good wettability and therefore, higher surface energies, than expected from the contact angle measurements. Differences in cytoskeletal organization between the superficial cellular layer and SSCs directly adherent on the template surface (Figure 3; Video S1 in Supplementary Material) indicate the crucial role played by the secreted extracellular matrix, as discussed in greater detail by Feller et al. (2015). Equally important is the paracrine effect of cells directly adherent on a scaffold surface on cells distal to the implant which has previously been shown to be effected by their secretion of PGE_2_, osteoprotegerin, and TGF-β1 (Lincks et al., 1998). *In vivo*, these local factors inhibit osteoclastic activity in addition to enhancing osteoblastic activity. This effect has been shown to be augmented by the presence of 1,25-VitD3 (Gail and Boone, 1970). Immobilizing such stimuli on the scaffold surface may be a potentially viable approach in enhancing the osseointegrative capability of titanium devices.

The high porosity of the 90-µm pore surface could limit the spread of the centrally deposited cell-laden droplet over the template surface, which in turn, could have contributed to the constraint of cellular migration observed. Furthermore, the rate of random cell migration has been implicated in generating a uniform cell population distribution and thus, a uniform coverage of a surface (Gail and Boone, 1970). The reduced surface area available would have limited the rate of random cell migration, as reflected by the limited distribution of the cells at day 21. Despite the observed cellular proliferation (Figure 6; Figure S6 in Supplementary Material), the cells failed to cover the entirety of the template surface, with the vast majority of cells remaining localized to the template center. This is in marked contrast to cells seeded on TCP, which migrated across the entire surface of the wells in basal and osteogenic conditions, achieving full confluency. This issue could be overcome by three-dimensional printing techniques, such as inkjet cell printing, which would allow for a more uniform, scaffold-wide distribution of cells. AM techniques have even been utilized to alternately print different biomaterials per layer, with the aim of creating discrete surface energy gradients across a bioconstruct scaffold, which may trigger different cellular activity and differentiation, making it possible to generate distinct tissue types within a single bioconstruct (Di Luca et al., 2016).

Seeded SSCs also grew into the 90-µm pores, in addition to bridging the pores, irrespective of cell density and the mode of sterilization. This is in contrast to findings by Xue et al. (2007) who found that a critical pore size of 200 µm or higher was necessary for osteoblastic precursor cell line ingrowth into pores. Hollander et al. (2006) had shown that 5 × 10^4^ human osteoblasts cultured on direct laser forming Ti6Al4V scaffolds with 700- and 1,000-µm pore sizes did not completely bridge the pores but developed a circular-shaped growth pattern along the rims of the pores. The same number of cells covered the entirety of the surface of scaffolds with 500-µm sized pores. Neither study was able to determine the extent of cell growth into the pores. In our study, confocal microscopy imaging was utilized to create a three-dimensional reconstruction of the templates, allowing the measurement of the depth of cell ingrowth within the pores. Although SSCs were able to grow 25 deep into the 90-µm sized pores, the cells were unable to penetrate the full 100-µm depth of the conical-shaped pores. This finding suggests that pore geometry, as well as pore size, is key determinants in the extent of pore bridging and cell ingrowth within porous scaffolds, thus highlighting the importance of scaffold design in the development of successful implants for bone tissue engineering.

Previous studies have indicated that osteoblasts are capable of discriminating micro-scale topographical features on titanium and titanium alloy surfaces, with increased osteogenic differentiation on surfaces containing micro-scale roughness (Lincks et al., 1998; Zhao et al., 2006; Cheng et al., 2014; Olivares-Navarrete et al., 2014). Olivares-Navarrette and colleagues demonstrated that MSCs developed osteoblastic characteristics when grown on micro-structured titanium surfaces, even in the absence of osteogenic medium supplements such as β-glycerophosphate and dexamethasone. This osteoinductive capability was more pronounced on surfaces that were more hydrophilic (Olivares-Navarrete et al., 2010). Zhao et al. demonstrated that MG63 cells develop more filopodia on rougher titanium surfaces compared to smooth surfaces (Zhao et al. 2007). Work recently published by Banik et al. further indicates that MSCs develop a fibroblastic morphology when seeded on smooth titanium surfaces, whereas on rough titanium surfaces, MSCs are mostly cuboidal or stellate in appearance (Banik et al. 2016). Such morphological changes have previously been shown in cells undergoing osteogenic differentiation (Dalby et al., 2007; Hong et al., 2010). Our results concur with these published data, with seeded SSCs demonstrating morphological, functional, and gene expression changes in keeping with early and persistent osteogenic differentiation in both the titanium and osteogenic media TCP groups. The osteoinductive effect of titanium on SSCs could further explain the lower proliferative rate of seeded SSCs compared to those cultured on TCP as previously reported (Lincks et al., 1998; Zhao et al., 2007; Banik et al., 2016).

## Conclusion

This study has demonstrated that laser-generated microporous, microrough titanium templates could facilitate rapid SSC adhesion and induce early osteogenic differentiation of SSCs despite the seeded surface being inverted and suspended throughout a 21-day culture period. Early osteogenic differentiation resulted in a lower proliferative capacity and surface migration of seeded SSCs, which subsequently left exposed areas on the template and could potentially contribute to poorer long-term osseointegration *in vivo*. This issue could be overcome through adequate cell seeding and placement utilizing three-dimensional printing techniques. Pore geometry and size appeared to affect the degree of cellular overgrowth within pores as SSCs only grew a distance of 25- to 100-µm deep conical pores, highlighting the critical effect of scaffold design on device functionality. Finally, the impact of the method of sterilization and storage on the surface properties of implants is crucial as part of quality assurance evaluation during the manufacturing process. In summary, the current study shows that microporous, microrough titanium could be used as a superficial template or bridging interface between an implant and the bone surface to enhance peri-implant bone wound healing and thus, osseointegration of the implant, while minimizing implant alloy-related wear debris toxicity and critically, in the longer term, improve implant safety, functionality, and longevity.

## Author Contributions

DT—designed and performed all experiments described within the paper, primary author of the manuscript. LYY and KLO—involved in design and data analysis and overall edit of the manuscript, involved in the provision of scaffolds described within the paper. RO—principal investigator who oversaw experiment design and progression, involved in the final editing process and data analysis of the draft manuscript.

## Conflict of Interest Statement

The authors declare that the research was conducted in the absence of any commercial or financial relationships that could be construed as a potential conflict of interest.

## Supplementary Material

Figure S1 This video demonstrates the biocompatibility of the titanium template while confirming the adhesion of human skeletal stem cells to the surface of the template despite an inverted culture process over 21 days. It also enables the visualisation of the different behaviour of cytoskeletal elements within the different cell layers as well as within the pores of the template over time.
