# Supplementary material for: Repositioning Titanium: An In Vitro Evaluation of Laser-Generated Microporous, Microrough Titanium Templates As a Potential Bridging Interface for Enhanced Osseointegration and Durability of Implants
Source: Front Bioeng Biotechnol. 2017 Dec 11;5:77. doi: 10.3389/fbioe.2017.00077 (PMC5732141; doi:10.3389/fbioe.2017.00077)
Supplement: Supplementary file 1 [file data_sheet_1.docx]

SUPPLEMENTARY INFORMATION

**Re-positioning titanium: an *in vitro* evaluation of laser-generated microporous, microrough titanium templates as a potential bridging interface for enhanced osseointegration and durability of implants.**

**Daniel Tang**^a^, **Liang-Yo Yang**^b, c, d^, **Keng-Liang Ou**^e, f, g, h*^, **Richard O.C. Oreffo**^a*^

^a^Centre for Human Development, Stem Cells and Regeneration, Faculty of Medicine, University of Southampton, Southampton SO16 6YD, United Kingdom

^b^Department of Physiology, School of Medicine, College of Medicine, China Medical University, Taichung 40402, Taiwan

^c^Research Center for Biotechnology, China Medical University Hospital, China Medical University, Taichung 40402, Taiwan

^d^Department of Biotechnology, College of Medical and Health Science, Asia University, Taichung 41354, Taiwan

^e^Department of Dentistry, Cathay General Hospital, Taipei 106, Taiwan

^f^Department of Dentistry, Taipei Medical University Hospital, Taipei 110, Taiwan

^g^Department of Dentistry, Taipei Medical University – Shuang Ho Hospital, New Taipei City 235, Taiwan

^h^3D Global Biotech Inc., New Taipei City 221, Taiwan

**Corresponding authors*:

**Professor Richard O.C. Oreffo**

Bone and Joint Research Group,

Centre for Human Development, Stem Cells & Regeneration,

Human Development and Health, Institute of Developmental Sciences,

University of Southampton, Southampton SO16 6YD, United Kingdom.

Telephone: +44 (0)2381 208502

Fax +44 (0)2381 205525

Email: roco@soton.ac.uk; www.stemcells.org.uk

**Professor Keng-Liang Ou**

3D Global Biotech Inc.,

New Taipei City 221, Taiwan.

Telephone: +886-2-26971270 ext. 100

Fax: +886-2-26971272

E-mail: klouyu@gmail.com

| Gene marker | Gene-specific primer sequence | | T_m_ (°C) | NCBI Reference Sequence |
| --- | --- | --- | --- | --- |
|  | *Forward* | *Reverse* |  |  |
| β-actin | *GGCATCCTCACCCTGAAGTA* | *AGGTGTGGTGCCAGATTTTC* | 58 | NM_001101.3 |
| Runx2 | *GTAGATGGACCTCGGGAACC* | *GAGGCGGTCAGAGAACAAAC* | 59 | XM_011514966.2 |
| Col1a1 | *GAGTGCTGTCCCGTCTGC* | *TTTCTTGGTCGGTGGGTG* | 59 | XM_005257059.4 |
| ALP | *GGAACTCCTGACCCTTGACC* | *TCCTGTTCAGCTCGTACTGC* | 60 | NM_000478.5 |
| OPN | *GTTTCGCAGACCTGACATCC* | *CATTCAACTCCTCGCTTTCC* | 58 | NM_001251830.1 |
| OCN | *AAGAGACCCAGGCGCTACCT* | *AACTCGTCACAGTCCGGATTG* | 62 | NM_199173.5 |
| TBP | *TGCACAGGAGCCAAGAGTGAA* | *CACATCACAGCTCCCCACCA* | 62 | NM_003194.4 |

**Table ‎S1** **Primers used for RT-qPCR** (*designed by May De Andres Gonzalez*). Sequences were cross-checked using Primer-BLAST (<http://www.ncbi.nlm.nih.gov/tools/primer-blast>).

| *Gene marker* | *Variable* | *F (DFn, DFd)* | *p value* | *h^2^* |
| --- | --- | --- | --- | --- |
| Runx2 | Time | F (2, 4) = 84.76 | < 0.001 | 0.231 |
|  | Approach | F (2, 4) = 97.34 | < 0.001 | 0.389 |
|  | Interaction | F (4, 8) = 181.7 | < 0.001 | 0.36 |
| Col1a1 | Time | F (2 ,4) = 1424 | < 0.001 | 0.822 |
|  | Approach | F (2 ,4) = 89.35 | < 0.001 | 0.096 |
|  | Interaction | F (4, 8) = 89.1 | < 0.001 | 0.076 |
| ALP | Time | F (2, 4) = 248.9 | < 0.001 | 0.263 |
|  | Approach | F (2 ,4) = 656.6 | < 0.001 | 0.348 |
|  | Interaction | F (4, 8) = 495.6 | < 0.001 | 0.383 |
| OPN | Time | F (2, 4) = 393.2 | < 0.001 | 0.265 |
|  | Approach | F (2, 4) = 441.5 | < 0.001 | 0.304 |
|  | Interaction | F (4, 8) = 638.4 | < 0.001 | 0.425 |
| OCN | Time | F (2, 4) = 260.1 | < 0.001 | 0.01 |
|  | Approach | F (2, 4) = 1844 | < 0.001 | 0.699 |
|  | Interaction | F (4, 8) = 299.4 | < 0.001 | 0.198 |
| β-actin | Time | F (2, 4) = 505.3 | < 0.001 | 0.783 |
|  | Approach | F (2, 4) = 115.5 | < 0.001 | 0.063 |
|  | Interaction | F (4, 8) = 101.8 | < 0.001 | 0.145 |

**Table S2** **Summary of two-way ANOVA test of the effects of culture time and culture approach on the expression of gene markers Runx2, Col1a1, ALP, OPN, OCN, and β-actin.** The complex, statistically significant interactions between culture time and culture approach make it difficult to clearly distinguish which variable is primarily responsible for the changes in gene expression observed.


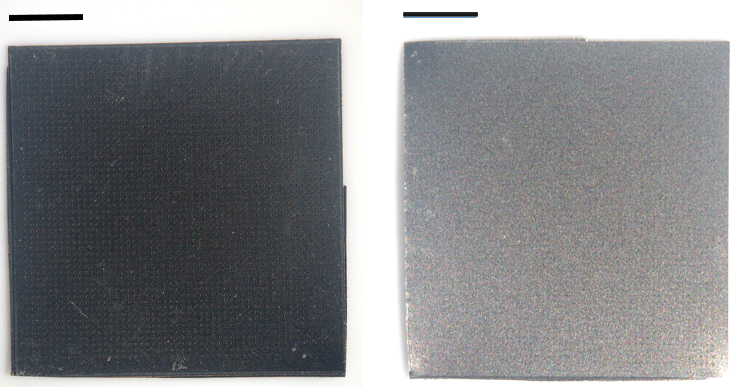


**Figure S1** *Left*: Photograph of the 90 µm pore surface of the titanium template. *Right*: Photograph of the 9 µm pore surface of the titanium template. Scale bars represents 200 µm.


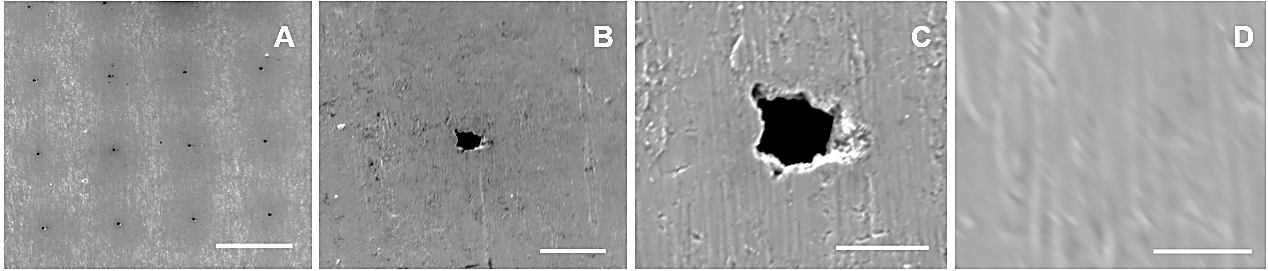


**Figure S2 Surface appearance of cell-free titanium templates using SEM.** A) Overview of the 9 µm pore surface (scale bar represents 200 µm). B) High magnification view of a 9 µm pore (scale bar represents 20 µm). C) Minimal surface roughness is observed in the immediate vicinity of the 9 µm pore (scale bar represents 10 µm). D) The interpore area of the 9 µm pore surface appears smoother than the corresponding area on the 90 µm pore surface (scale bar represents 4 µm).


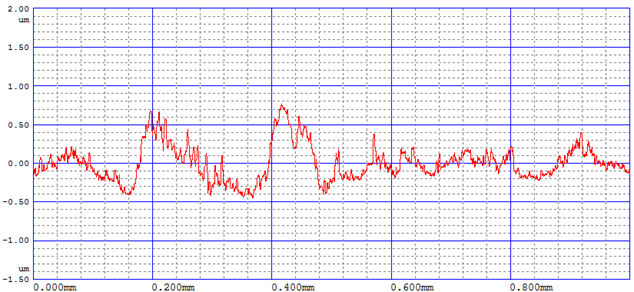

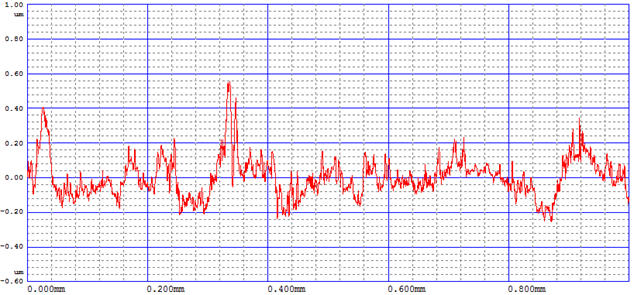


**B**

**A**


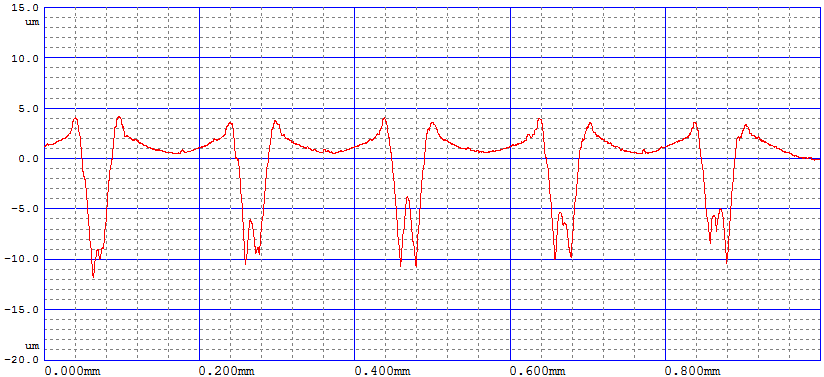

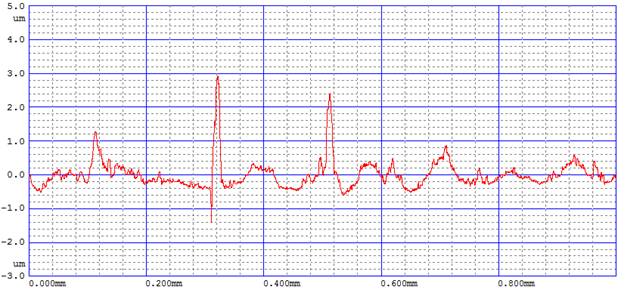


**D**

**C**

**Figure S3 Surface roughness measurements.** Surface roughness was determined over a 1 mm length over 3 separate areas of A) the surface of a medical grade IV titanium sheet prior to generation of 90 µm-sized pores, B) the surface of a medical grade IV titanium sheet prior to the generation of 9 µm-sized pores, C) the 90 µm pore surface of an EOS titanium template (black arrows indicate the interpore areas), and D) the 9 µm pore surface of an EOS titanium template.


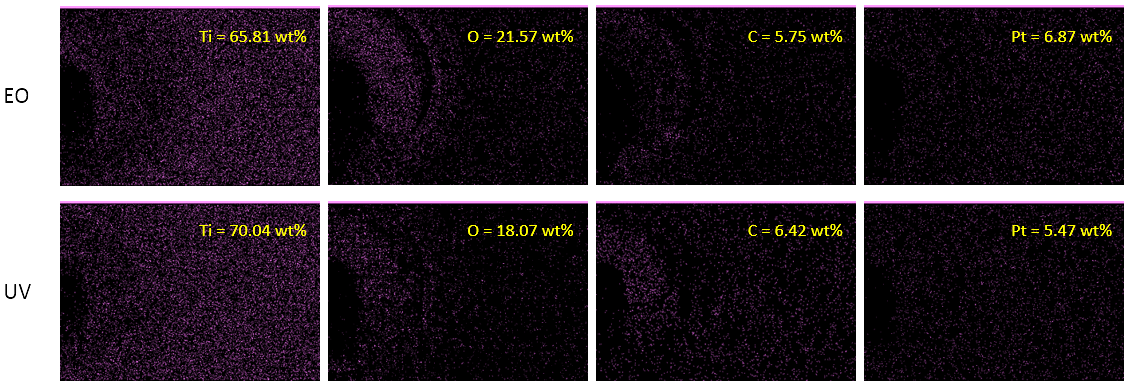


**Figure S4 Surface mapping by EDS of EOS and UV titanium templates.** EOS templates demonstrated a higher overall weight percent oxygen content when compared to UV templates. Surface mapping (false-coloured magenta) revealed that oxygen distribution was primarily localised to the pore area of the 90 µm pore surface, with a brighter signal intensity in EOS pore areas rather than UV pore areas. This observed difference in oxygen content could be related to the oxidising effect of ethylene oxide gas.


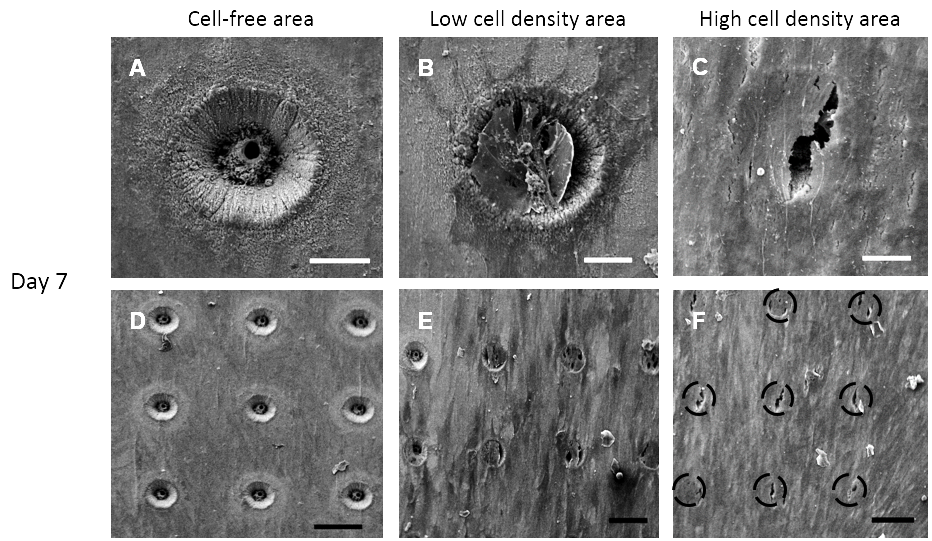


**Figure S5 SEM images of SSC-seeded EOS titanium templates at day 7**. A) 90 µm pore without cells (scale bar represents 35 µm). B to C) 90 µm pore appearance as cell density increases (scale bar represents 30 µm ). D) 90 µm pore surface without cells (scale bar represents 110 µm). E to F) 90 µm pore surface appearance as cell density increases (scale bar represents 110 µm). Black dashed circles demarcate the 90 µm pores.


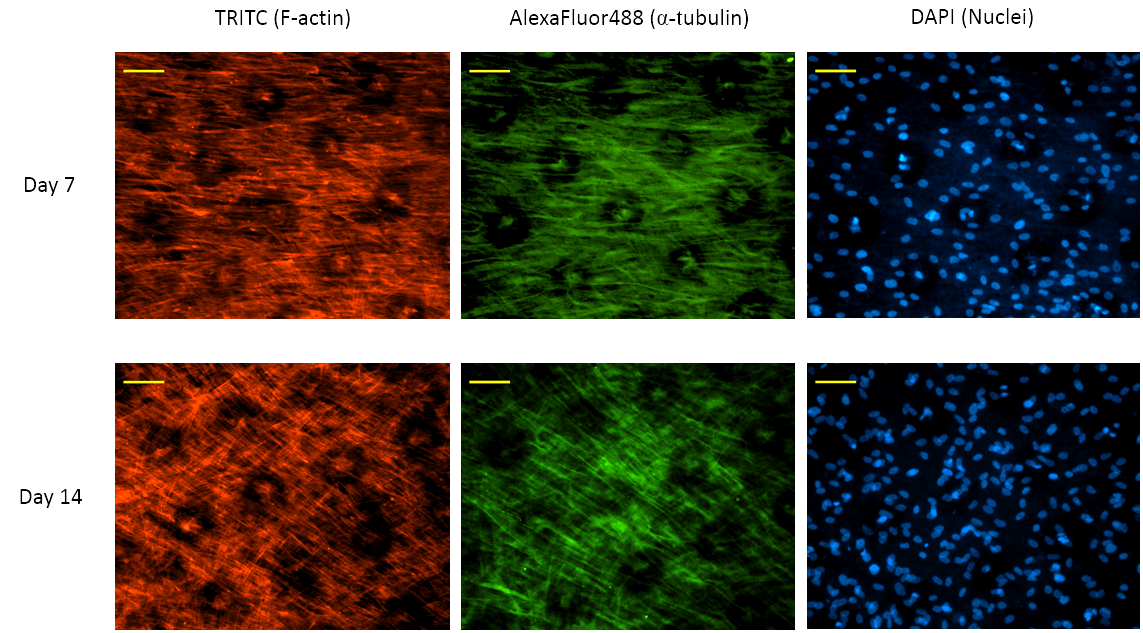


**Figure S6 SSC cytoskeletal and nuclei changes over time on EOS titanium templates.** F-actin (red) and α-tubulin (green) filament orientation appears organised by day 7, with a concomitant increase in signal intensity by day 14 as cell density increased due to SSC proliferation over time, as demonstrated by the increase in nuclei numbers (blue). Scale bars represent 100 µm.


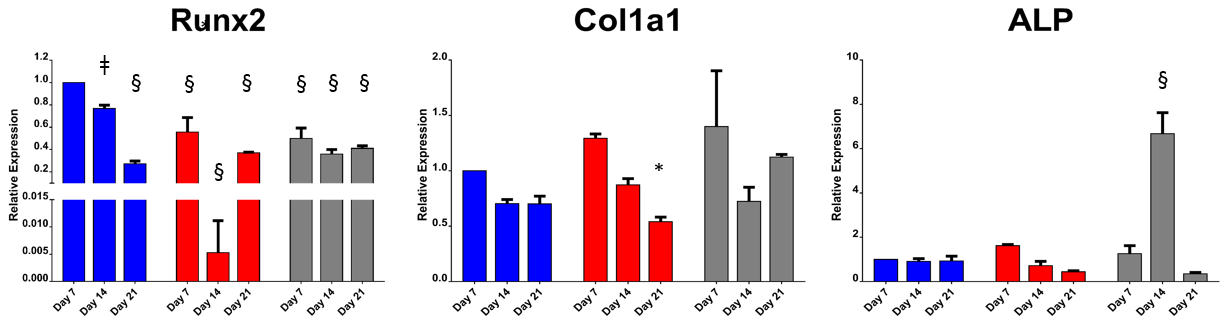


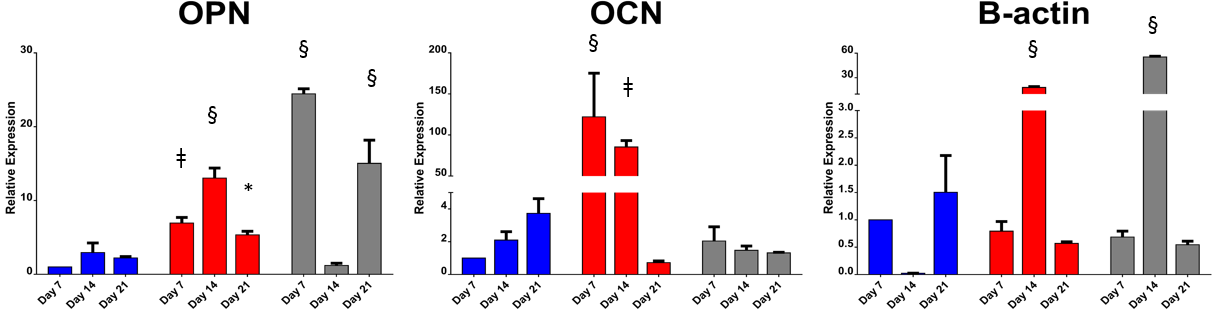


**Figure S7 Relative expressions of osteogenic differentiation markers Runx2, Col1a1, ALP, OPN, and OCN, and the cytoskeletal marker, β-actin, at days 7, 14, and 21.** SSCs seeded on TCP were cultured in basal (in blue) and osteogenic (in red) media while those on UV titanium templates were cultured in basal media (in grey). SSCs cultured on UV templates demonstrated less persistent osteogenic differentiation. This difference could be a consequence of the more hydrophobic surface of UV templates resulting in poorer cell adhesion to the template surface. (* = *p* < 0.05, ǂ = *p* < 0.01, § = *p* < 0.001.)


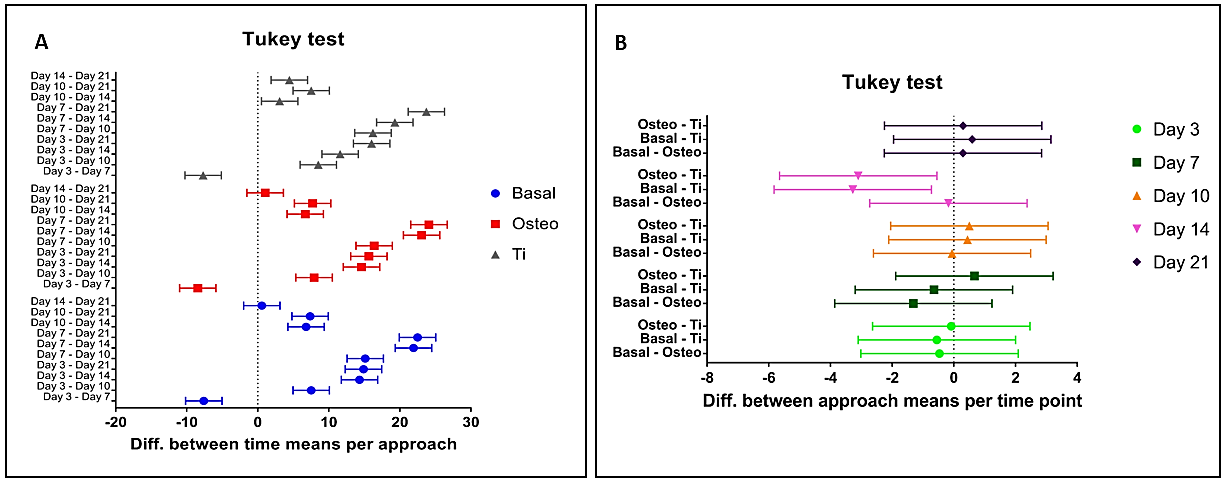


**Figure S8 Tukey *post-hoc* test to determine the significance of the individual effects on ALP activity.** A) Culture time had significant effects within each culture approach, with all comparisons demonstrating extremely significant (*p* < 0.001) changes in ALP activity, except for day 14 compared to day 21 in basal and osteogenic groups (*p* > 0.05) and day 10 compared to day 14 in the EOS titanium group (*p* = 0.015). B) Culture approach however, did not demonstrate significant effect on ALP activity at each time point, except at day 14 in the basal-titanium (*p* = 0.01) and osteogenic-titanium (*p* = 0.015) groups. Culture time therefore plays a crucial role in changes in ALP activity.
